# Comprehensive Care through Family Medicine: Improving the Sustainability of Aging Societies

**DOI:** 10.3390/geriatrics6020059

**Published:** 2021-06-04

**Authors:** Ryuichi Ohta, Akinori Ueno, Jun Kitayuguchi, Yoshihiro Moriwaki, Jun Otani, Chiaki Sano

**Affiliations:** 1 Community Care, Unnan City Hospital, Unnan 699-1221, Shimane, Japan; yoshimoriwaki@gmail.com (Y.M.); blackjack.otani@nifty.ne.jp (J.O.); 2 Unnan Public Health Center, Unnan 699-1311, Shimane, Japan; ueno-akinori@pref.shimane.lg.jp; 3 Physical Education and Medicine Research Center Unnan, Unnan 699-1105, Shimane, Japan; junk_907@yahoo.co.jp; 4 Department of Community Medicine Management, Faculty of Medicine, Shimane University, Izumo 693-8501, Shimane, Japan; sanochi@med.shimane-u.ac.jp

**Keywords:** family medicine, sustainability, comprehensive care, medical care, rural medicine, older people, aging society, health outcome, Japanese, general physician

## Abstract

Comprehensive care through family medicine can enhance the approach to multimorbidity, interprofessional collaboration, and community care, and make medical care more sustainable for older people. This study investigated the effect of implementing family medicine and the comprehensiveness of medical care in one of the most rural communities. This implementation research used medical care data from April 2015 to March 2020. Patients’ diagnoses were categorized according to the 10th revision of the International Statistical Classification of Disease and Related Health Problems (ICD-10). In 2016, family medicine was implemented in only one general hospital in Unnan. The comprehensiveness rate improved in all ICD-10 disease categories during the study period, especially in the following categories—infections; neoplasms; endocrine, nutritional, and metabolic diseases; mental disorders; nervous system; circulatory system; respiratory system; digestive system; skin and subcutaneous tissue; musculoskeletal system and connective tissue; and the genitourinary system. Implementing family medicine in rural Japanese communities can improve the comprehensiveness of medical care and resolve the issue of fragmentation of care by improving interprofessional collaboration and community care. It can be a solution for the aging of both patient and healthcare professionals. Future research can investigate the relationship between family medicine and patient health outcomes for improved healthcare sustainability.

## 1. Introduction

Comprehensive care is vital in an aging society to holistically support a variety of health problems and develop a sustainable medical care system [1]. To ensure comprehensive care, approaches to multimorbidity, interprofessional collaboration (IPC), and community care should be enhanced. Regarding multimorbidity, aging causes various health problems, which can decrease the quality of life (QOL) [2]. Such medical problems cannot be solved solely by healthcare professionals, because the problems can be connected to psychosocial problems, which reinforce the need for IPC [3]. To approach a multitude of health problems, various healthcare professionals must collaborate [1,3]. Medical professionals must comprehensively and effectively deal with multimorbidity, and care professionals need multiple skills to take care of older people for them to continue their lives in their homes; this is done by collaborating with their families and social support systems [4]. Furthermore, medical and care professionals can collaborate with each other to manage older patients with various biopsychosocial problems and multimorbidity [5,6]. Regarding community care, citizens in communities must also enhance their abilities to deal with their health problems by using social support and social capital [7]. Governments and healthcare professionals can support communities by improving their abilities financially and socially [8,9]. As the world population continues to age, the importance of comprehensive care should be reinforced to facilitate the sustainability of health care, and it should be further investigated for better application.

In Japan, the percentage of the population above the age of 65 years is the largest in the world [10]. Thus, all stakeholders must contribute to the establishment of primary health care to ensure a sustainable healthcare system [1]. However, the free access system of Japanese medical care impinges on the establishment of comprehensive care [11]. Patients in Japan can go to any medical institution if they exhibit symptoms. Although the government has implemented various policies, the proportion of patients who go directly to general or specialized hospitals is high, leading to the exhaustion of physicians in those hospitals. Moreover, the free access system can deteriorate the transition of care from hospitals to clinics and communities, especially in rural areas, where older patients tend to visit hospitals and clinics in nearby urban areas for specialist care [12,13]. When these patients have acute symptoms or become dependent, they cannot go to urban areas and are limited to the medical institutions near their homes. As there is limited information in these medical institutions, they may not receive appropriate medical care; this phenomenon is called fragmentation of care [14]. As patients in rural areas are old and dependent, healthcare institutions must collaborate and accommodate patients, which will mitigate the fragmentation of care [15,16]. Rural medical institutions must improve their collaboration and rebuild their reputations, as some patients hesitate to go there due to a lack of collaborative care [17]. To ensure the sustainability of health care, the establishment of comprehensive care should also be contextualized in each setting [18]. The speed of aging and the decrease in the population are more rapid in rural than in urban areas, and robust comprehensive care is needed to support people in such areas to maintain their QOL [19].

As a medical specialist dealing with multimorbidity, IPC, and community care, a specialty of family medicine has been created in Japan to improve the comprehensiveness of medical care. The competency of family medicine in Japan includes that of family medicine and general practitioners and works in various settings, such as urban or rural hospitals and clinics [20]. Physicians that specialize in family medicine are called family physicians and deal with multiple medical problems by collaborating with other specialists. For comprehensive care, they collaborate with various healthcare professionals to care for patients in communities and motivate patients to promote their health as community care [20]. This specialty can improve the condition of comprehensive care and reduce the fragmentation of medical care. Therefore, our research question was: “Does the application of family medicine in communities improve the comprehensiveness of medical care?” Family medicine should be utilized to improve comprehensive care, and the effectiveness of family medicine in improving such care should be investigated. As comprehensive care provision depends on the context of communities, the implementation of family medicine should respect the context in which it is being provided. For better evidence of family medicine, evidence from different contexts is required. In this study, our aim was to investigate the change in comprehensiveness of medical care through the implementation of family medicine in rural communities.

## 2. Materials and Methods

### 2.1. Design

This study was an implementation research study with patients who lived in Unnan City, Japan. We used medical care data from April 2015 to March 2020 to investigate the change in comprehensiveness of medical care in rural areas by implementing family medicine in April 2016.

### 2.2. Setting

Unnan City, which is located in the southeast of Shimane Prefecture, is one of the most rural areas in Japan. In 2020, the total population of Unnan was 37,638 (18,145 males and 19,492 females), with 39% being over 65 years old; this proportion is expected to reach 50% by 2025 [9]. Furthermore, Unnan has one of the lowest densities with respect to the number of physicians per 1000 people in Japan [9]. It contains 16 clinics, 12 home care stations, 3 visiting nurse stations, and 1 public hospital (i.e., Unnan City Hospital). In 2019, the hospital staff comprised 27 physicians, 197 nurses, 7 pharmacists, 15 clinical technicians, 37 therapists (22 physical therapists, 12 occupational therapists, and 3 speech therapists), 4 nutritionists, and 34 clerks. Full-time physicians practiced in the following departments: internal medicine, surgery, orthopedics, pediatrics, dermatology, urology, otolaryngology, obstetrics and gynecology, and family medicine. No other medical institutions in Unnan had a recovery rehabilitation unit [21]. In the study duration, the number of general physicians increased by three. Unnan City is next to two urban areas: Matsue City and Izumo City. Based on the Japan Medical Analysis Platform, Matsue had 11 hospitals, 180 clinics, and 540 physicians, while Izumo had 11 hospitals, 144 clinics, and 811 physicians [22]. Unnan residents can reach hospitals and clinics in Matsue and Izumo after a 30-min drive. As the Japanese medical system allows free access to medical institutions for all citizens, patients can choose any medical institution based on their needs and demands.

### 2.3. Participants

Patients who lived in Unnan City, Japan, and availed medical care in the city from 1 April 2015 to 31 March 2020, were included in this study.

### 2.4. Family Medicine in Unnan City

**Figure 1:**
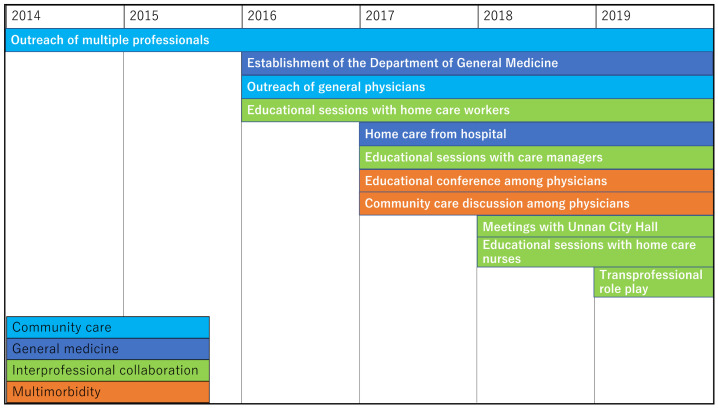
Approaches to family medicine within Unnan City Hospital.

In Unnan City Hospital, multiple approaches were adopted to address the categories of multimorbidity, IPC, and community care (Figure 1).

#### 2.4.1. Approach to Multimorbidity

Rural medical physicians are old and struggle to manage various medical problems in hospitals and clinics. Research has shown that between 1994 and 2014, the average age difference of physicians in urban and rural areas widened by 4.9%, and the number of older physicians (aged 55–70 years) increased by 153% [23]. In Unnan City, clinic and hospital physicians have discussed medical care since April 2017. Once every four months, they discuss medical issues regarding their difficulties at work. The increased medical dependence and multimorbidity of patients, being bound to rural areas, and patients’ loyalty to primary care physicians were found to exhaust some physicians [24]. To establish comprehensive care, their burden should be alleviated. A solution to improve task-shifting, hospital-clinic relationships, and IPC should be implemented [24]. In 2016, Unnan City Hospital established a Department of Community Care specializing in family medicine. This feature was implemented by accepting all consultations from clinical physicians and managing patients’ multimorbidity in admissions. General physicians encourage clinical physicians to consult their patients in the hospital through regular discussions. Furthermore, to address the problem of multimorbidity in the city and improve IPC among physicians, educational conferences were held every three months to improve the knowledge and skills of the physicians in Unnan City. In the conferences held, the average number of participants was 14. A conference regarding community care among clinical and hospital physicians was held once every four months to improve mutual understanding among them. In such conferences, the average number of participants was seven.

#### 2.4.2. Approach to Interprofessional Collaboration (IPC)

To improve the IPC in Unnan City, healthcare professionals’ perceptions regarding IPC have been inquired into since 2016. Through discussions with home care nurses, home care workers, care managers, and physicians, the misunderstandings and lack of humanistic relationships among healthcare professionals were clarified. Home care nurses have various conflicts with care professionals, local governments, and nurses from healthcare institutions, in addition to conflicts with physicians due to professional hierarchy. The conflicts between hospital nurses and home care nurses may be intense due to misunderstandings in clinical settings [25]. Home care professionals—especially home care workers and care managers—are often contacted by patients for healthcare services at home [26]. Thus, they can experience difficulties in dealing with the acute conditions of providing home care [27,28]. They also struggle with information-sharing among medical and home care professionals [27,28]. Home care workers have a process to convey the conditions of patients to other professionals, as well as patients’ families. Care managers play a vital role in this process. As the background of most care managers is in the care profession, they do not have enough medical knowledge to judge the changes in the conditions of patients receiving home care [28]. The same can be applied to home care workers. Home care workers encounter various acute symptoms in patients receiving home care. Research from Unnan City shows that 16% of patients receiving home care may have acute symptoms, over 90% of home care patients need medical attention, and 46% of them need admission [26]. Based on grounded theory research from Unnan City, home care workers contract the mild symptoms of patients receiving home care, and when they get worse, they share the information with care managers and medical professionals [29]. Effective information sharing and collaboration may enhance patient care in Unnan City. Thus, to improve care managers’ and home care workers’ understanding of medical care, educational conferences were held by the general physician of Unnan City Hospital twice a year, with the number of participants in each conference, ranging from 40 to 55. Moreover, to improve the understanding and humanistic relationships among healthcare professionals, trans professional role-play was facilitated by general physicians in the city. In the role-play, each professional played a role that was different from their original profession in a case conference. Through this activity, healthcare professionals realized their competency in IPC, improved their understanding of other professionals, and formed humanistic relationships with each other [30]. Role-play was performed once per year.

#### 2.4.3. Approach to Community Care

**Figure 2:**
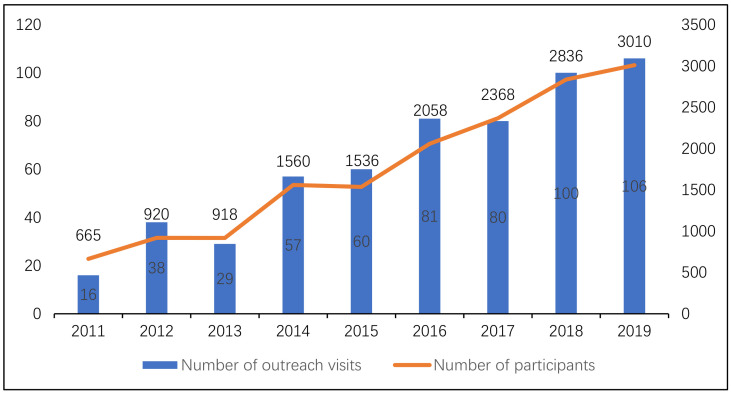
Number of outreach visits and number of participants during each visit.

General physicians have investigated the difficulties faced by residents of Unnan City regarding health conditions since 2016. Research has shown that residents of Unnan City have difficulty managing their health conditions due to a lack of effective communication with healthcare professionals [19]. The availability of medical care may decrease, not only due to patients’ aging, but also due to the aging of medical and care professionals. The number of medical and care professionals has gradually decreased, and there is a decline in the number of clinics due to the retirement of older physicians [17]. Residents of rural areas are anxious about their poor help-seeking behaviors and health maintenance, which are caused by a lack of understanding of their medical conditions. The lack of knowledge about their health conditions and inappropriate help-seeking behaviors can worsen their health conditions, possibly resulting in overdiagnosis and overtreatment [17]. Since 2011, various medical professionals have visited community centers to teach essential knowledge and skills for maintaining the health conditions of residents of rural areas. To improve such conditions, general physicians have educated residents regarding help-seeking behaviors [31]. After the implementation of family medicine, the number of outreach initiatives of medical professionals to such communities has increased, improving the community care provided by hospitals (Figure 2).

### 2.5. Measurements

#### 2.5.1. Demographic Data of Patients

The demographics of citizens of Unnan City were collected from the Unnan City citizen database. Anonymous patient data were extracted from the Shimane Prefecture insurance system. The duration of the collection of data was from April 2015 to March 2020. The extracted data were age, sex, medical diagnosis at admission and outpatient departure, and the places where patients availed medical care in Japan. The diagnoses of the patients were categorized based on the 10th revision of the International Statistical Classification of Disease and Related Health Problems (ICD-10). The places where medical care was availed were delimited as Unnan City and areas outside Unnan City. We used the categories in the ICD-10 to classify which diseases both specialty and general physicians can care for in Unnan City Hospital: I—certain infectious and parasitic diseases; II—neoplasms; III—diseases of the blood and blood-forming organs and certain disorders involving the immune mechanism; IV—endocrine, nutritional, and metabolic diseases; V—mental and behavioral disorders; VI—diseases of the nervous system; IX—diseases of the circulatory system; X—diseases of the respiratory system; XI—diseases of the digestive system; XII—diseases of the skin and subcutaneous tissue; XIII—diseases of the musculoskeletal system and connective tissue; and XIV—diseases of the genitourinary system.

#### 2.5.2. The Rate of Comprehensiveness as a Primary Outcome

To measure the comprehensiveness of medical care in Unnan City, we defined the rate of comprehensiveness as the primary outcome. The rate of comprehensiveness was calculated as the number of patients who availed medical care in Unnan City divided by the total number of patients who availed medical care. Patients who availed medical care in both Unnan City and other places were counted in both categories. The rate of comprehensiveness was computed for each year in the study duration and each category in ICD-10. The rate of comprehensiveness can reveal the extent to which Unnan City could provide comprehensive medical care to patients.

### 2.6. Statistical Analysis

**Figure 3:**
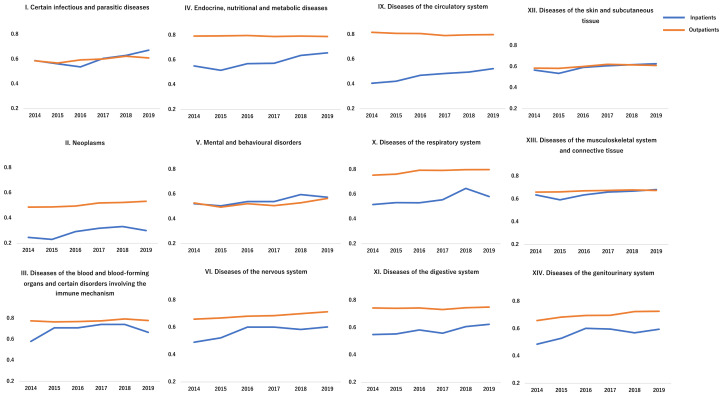
Changes in the rate of comprehensiveness during the study period.

The demographic data of the population and patients in Unnan City are shown in Table 1. The data pertaining to the ICD-10 categorization of diseases, as well as the rate of comprehensiveness, are shown descriptively (Table 2) and graphically (Figure 3) to illustrate the change in data throughout the study duration. Chi-squared tests were performed on the data obtained between 2015 and 2019, to examine the changes in the comprehensiveness of medical care. The statistical significance was set at *p* < 0.05.

### 2.7. Ethical Approval

The anonymity and confidentiality of patients’ information were ensured throughout the study. Only anonymous data were provided by the Unnan Public Health Center. The research information was posted on the hospital website without including any patient information. The contact information of the hospital representative was likewise listed on the website so that questions about the research could be answered at any time. All procedures included in this study were performed in compliance with the Declaration of Helsinki and its subsequent amendments. The Unnan City Hospital Clinical Ethics Committee approved the study protocol (No. 20200026).

## 3. Results

The Unnan City population has gradually decreased and aged since 2014. In both inpatient and outpatient categories, the rate of patients under 64 years of age has gradually decreased, and the total number of patients fluctuated throughout the study period among both the groups—men and women (Table 1).

The rate of comprehensiveness significantly increased among both the inpatient and outpatient departures during the study period (Table 2). The increases were statistically significant in the following categories after the implementation of family medicine in 2016: (1) certain infectious and parasitic diseases, neoplasms, diseases of the circulatory system, in both inpatient and outpatient departures; (2) endocrine, nutritional, and metabolic diseases, diseases of the digestive system, diseases of the musculoskeletal system and connective tissue in the inpatient departure; (3) mental and behavioral disorders, diseases of the nervous system, diseases of the respiratory system, diseases of the skin and subcutaneous tissue, and diseases of the genitourinary system in the outpatient departure. Regarding outpatient departure, the rate of comprehensiveness was consistent throughout the duration of the study (Figure 3). In all categories, the gap between the rates of outpatient departure and admission narrowed during the study period.

## 4. Discussion

This study showed that the implementation of family medicine in rural Japanese communities could improve the comprehensiveness of medical care provided. Due to the approaches to multimorbidity, IPC, and community care in the community hospital, consultations from communities and clinics to the community hospital may increase—increasing the rate of comprehensiveness of medical care in the rural community. However, despite four years of family medicine provision, the gap between the rate of comprehensiveness between outpatients and inpatients remained wide in several ICD-10 disease categories, even though all the gaps gradually narrowed. Thus, approaches for narrowing these gaps should be taken in the future.

Improvements in comprehensiveness in admission were prominent in the categories wherein few specialists were in rural Japan. In this study, notable improvements in inpatient comprehensiveness were observed in the following ICD-10 categories: certain infectious and parasitic diseases; endocrine, nutritional, and metabolic diseases; diseases of the circulatory system; and diseases of the nervous system. According to statistics in Shimane, Japan, the rates of specialists were 0.4% in infectious diseases, 1.6% in endocrine diseases, 7% in cardiovascular diseases, and 4.4% in neurology, all of which are lower than the 30.8% in general internal medicine, 10.6% in gastroenterology, and 10.3% in pediatrics. Family medicine as a medical specialty can accommodate a variety of patients with multimorbidity that require special care [20]. The results showed that family medicine could be a solution to the lack of specialists in rural areas, especially in aged societies. Among older patients, the problems of multimorbidity with polypharmacy are prevalent, and comprehensive care is critical for their safety, QOL, and end of life care [32,33,34]. Future studies can investigate the effectiveness of family medicine regarding patient’s QOL in multiple settings by adding ICD-11 categories which is revised one of ICD-10.

General physicians could take care of not only various kinds of hospital patients, but also many other patients. In this study, although only three general physicians had started working in Unnan City, there was an increase in the number of admitted patients by 441 (i.e., 147 for every general physician). This number is relatively higher than the patients accommodated by other kinds of physicians, indicating that general physicians can accommodate more patients than other specialists in community hospitals—which can be a solution to the lack of physicians in rural areas. Our research was performed in a rural area (close to urban areas) that has one of the lowest physician/patient ratios, which might cause fragmentation of care [14,15]. To prepare for an aged society worldwide, increasing the number of general physicians could be vital to sustaining medical care for older patients with multimorbidity [35,36]. In other countries, family medicine education has been established, and this has dealt with the issues of multimorbidity and fragmentation of care [37]. However, although the implementation of family medicine education is expanding, its implementation is limited in rural settings [38]. Therefore, appropriate education and allocation of general physicians in rural community hospitals should be enforced by central and local governments as a critical policy.

This study shows a gap between the rates of comprehensiveness of inpatient and outpatient departments, the trend being especially prominent in the following disease categories: the circulatory system, respiratory system, digestive system, and neoplasms. As Figure 3 shows, the gap is narrowing in all disease categories. This condition is due to the increased accommodation of patients with multimorbidity, improvement of IPC, and the reliance of citizens on the community hospital. Nonetheless, diseases that require particular interventions, such as surgeries and percutaneous cardiovascular interventions, should be dealt with by general hospitals. To mitigate the fragmentation of care and ensure smooth transitions of treatment, collaboration between community hospitals and general hospitals is essential [39]. For better collaboration, the reliance on general physicians from other specialties should be enhanced. Family medicine has been established in other countries, and family physicians have authentic roles in medical care that suit different clinical contexts [40,41]. Other specialists and healthcare workers respect family physicians’ positions, which facilitate IPC [42,43,44]. However, in the Japanese context, there is a significant lack of proper education and information among specialists about general physicians [45]. This situation could be caused by the lack of evidence regarding family medicine and the fact that it is an emerging specialty in the medical field. To address the need for medical care, general physicians in rural areas should broaden the scope of their practice and accommodate various patients by collaborating with other specialists and healthcare workers. There is emerging evidence that interprofessional collaboration and comprehensive care can mitigate the fragmentation of care [46,47]. Because family medicine is essential for the sustainability of medical care in aged societies, it is necessary to collect new evidence regarding family medicine, mainly focusing on patients’ and citizens’ quality of care and health outcomes.

This study has several limitations. First, as the study design involves implementation research with a historical comparison, we could not show a cause-and-effect relationship. Another significant limitation of the study was the low number of participants and observation timings. Thus, future studies can use extended durations with interrupted time series analysis. Additionally, as this study focused only on the comprehensiveness of medical care, it did not show the effectiveness of family medicine in improving patients’ and citizens’ health outcomes in communities. Previous studies have suggested that comprehensiveness through family medicine has a significant effect on patient satisfaction and medical cost; however, research on the relationship between comprehensive care and health outcomes is scant [43,48,49]. Thus, future research should investigate this relationship. Finally, as this research was performed in a rural Japanese setting, its generalizability is limited. However, the description of rural family medicine can be applied to other rural locations. To improve the generalizability of findings regarding the effect of the implementation of family medicine, future studies can be performed in various settings with different medical care resources.

## 5. Conclusions

The implementation of family medicine in rural Japanese communities can improve the comprehensiveness of medical care through improvements in care of multimorbidity, IPC, and community care. Future research can investigate the relationship between family medicine and patient health outcomes for improving the sustainability of healthcare practices.

## Figures and Tables

**Table 1:** Demographics of Unnan City residents and patients.

	2015		2016		2017		2018		2019	
	Men	Women	Men	Women	Men	Women	Men	Women	Men	Women
**Total population**	19,535	21,229	19,309	20,863	19,027	20,587	18,720	20,162	18,384	19,736
Over 65 years old (%)	35.4		36.09		37.04		37.75		38.49	
Patients, *n* (%)										
Inpatients										
0–64	204 (12.1)	169 (8.7)	203 (12.1)	143 (7.5)	198 (11.6)	137 (7.3)	162 (9.9)	127 (6.9)	155 (9.1)	125 (6.9)
65–69	225 (13.4)	125 (6.5)	239 (14.3)	118 (6.2)	209 (12.3)	116 (6.1)	189 (11.6)	113 (6.2)	192 (11.3)	113 (6.2)
70–74	193 (11.5)	145 (7.5)	204 (12.2)	144 (7.6)	224 (13.2)	150 (7.9)	244 (14.9)	158 (8.6)	263 (15.5)	142 (7.8)
75–79	273 (16.3)	280 (14.5)	287 (17.1)	257 (13.5)	279 (16.4)	249 (13.2)	293 (17.9)	238 (13.0)	276 (16.2)	232 (12.8)
80–84	365 (21.7)	386 (20.0)	312 (18.6)	378 (19.8)	302 (17.7)	378 (20.0)	266 (16.3)	331 (18.0)	294 (17.3)	328 (18.1)
85–89	256 (15.2)	400 (20.7)	271 (16.2)	416 (21.8)	307 (18.0)	384 (20.3)	273 (16.7)	389 (21.2)	280 (16.5)	391 (21.6)
90+	164 (9.8)	427 (22.1)	160 (9.5)	451 (23.6)	184 (10.8)	473 (15.1)	207 (12.7)	480 (26.1)	242 (14.2)	479 (26.5)
Total	1680	1932	1676	1907	1703	1887	1634	1836	1702	1810
Outpatients										
0–64	1916 (25.8)	1954 (20.3)	1765 (24.2)	1771 (18.9)	1613 (22.7)	1587 (17.4)	1438 (20.8)	1463 (16.4)	1356 (19.7)	1385 (15.8)
65–69	1260 (17.0)	1200 (12.5)	1247 (17.1)	1137 (12.1)	1134 (15.9)	1041 (11.4)	1042 (15.1)	961 (10.8)	966 (14.0)	889 (10.2)
70–74	876 (11.8)	956 (9.9)	920 (12.6)	979 (10.4)	1017 (14.3)	1055 (11.6)	1124 (16.3)	1140 (12.8)	1233 (17.9)	1193 (13.6)
75–79	1196 (16.1)	1485 (15.4)	1185 (16.3)	1430 (15.2)	1175 (16.5)	1356 (14.9)	1158 (16.7)	1383 (15.5)	1166 (17.0)	1367 (15.6)
80–84	1105 (14.9)	1604 (16.6)	1063 (14.6)	1550 (16.5)	989 (13.9)	1531 (16.8)	936 (13.5)	1384 (15.5)	929 (13.5)	1299 (14.8)
85–89	705 (9.5)	1316 (13.7)	722 (9.9)	1308 (13.9)	783 (11.0)	1304 (14.3)	773 (11.2)	1319 (14.8)	753 (10.9)	1336 (15.3)
90+	372 (5.0)	1122 (11.6)	387 (5.3)	1206 (12.9)	410 (5.8)	1256 (13.8)	444 (6.4)	1270 (14.2)	476 (6.9)	1279 (14.6)
Total	7430	9637	7289	9381	7121	9130	6915	8920	6879	8748

**Table 2:** ICD-10 categories and rates of comprehensiveness.

	Inpatients						Outpatients					
	2015	2016	2017	2018	2019	*p*-Value	2015	2016	2017	2018	2019	*p*-Value
Certain infectious and parasitic diseases												
Total number	148	162	195	178	186	0.0412	1978	1965	1905	1873	1878	0.010
In the city	83	87	118	112	125		1123	1166	1144	1167	1144	
Rate of comprehensiveness	0.561	0.537	0.605	0.629	0.672		0.568	0.593	0.601	0.623	0.609	
Neoplasms												
Total number	524	558	569	541	542	0.0104	3174	3178	3431	3485	3490	<0.001
In the city	121	163	182	180	163		1553	1577	1779	1827	1859	
Rate of comprehensiveness	0.231	0.292	0.320	0.333	0.301		0.489	0.496	0.519	0.524	0.533	
Diseases of the blood and blood-forming organs and certain disorders involving the immune mechanism												
Total number	79	69	89	62	75	0.605	569	650	686	710	613	0.627
In the city	56	49	66	46	50		436	500	532	564	478	
Rate of comprehensiveness	0.709	0.710	0.742	0.742	0.667		0.766	0.769	0.776	0.794	0.780	
Endocrine, nutritional, and metabolic diseases												
Total number	287	265	264	283	289	<0.001	4558	4665	4572	4500	4606	0.589
In the city	148	151	151	180	190		3619	3716	3607	3567	3635	
Rate of comprehensiveness	0.516	0.570	0.572	0.636	0.657		0.794	0.797	0.789	0.793	0.789	
Mental and behavioral disorders												
Total number	188	187	226	213	226	0.166	1282	1305	1294	1332	1292	<0.001
In the city	95	101	122	127	130		635	683	655	704	730	
Rate of comprehensiveness	0.505	0.540	0.540	0.596	0.575		0.495	0.523	0.506	0.529	0.565	
Diseases of the nervous system												
Total number	256	271	271	257	247	0.073	1682	1826	1744	1735	1694	0.005
In the city	134	163	163	150	149		1125	1246	1197	1215	1210	
Rate of comprehensiveness	0.523	0.601	0.601	0.584	0.603		0.669	0.682	0.686	0.700	0.714	
Diseases of the circulatory system												
Total number	732	755	796	714	786	<0.001	7174	7175	7017	6972	6954	0.050
In the city	310	355	386	354	412		5805	5788	5548	5557	5534	
Rate of comprehensiveness	0.423	0.470	0.485	0.496	0.524		0.809	0.807	0.791	0.797	0.796	
Diseases of the respiratory system												
Total number	451	392	396	371	339	0.193	4647	4759	4580	4428	4191	<0.001
In the city	240	208	220	240	197		3541	3780	3626	3535	3349	
Rate of comprehensiveness	0.532	0.531	0.556	0.647	0.581		0.762	0.794	0.792	0.798	0.799	
Diseases of the digestive system												
Total number	577	583	589	555	564	0.016	4347	4181	3889	3781	3727	0.33
In the city	319	340	329	337	352		3221	3111	2847	2816	2797	
Rate of comprehensiveness	0.553	0.583	0.559	0.607	0.624		0.741	0.744	0.732	0.745	0.750	
Diseases of the skin and subcutaneous tissue												
Total number	140	123	130	134	148	0.121	2904	3018	3088	3119	3102	0.035
In the city	75	73	79	83	93		1696	1817	1922	1923	1895	
Rate of comprehensiveness	0.536	0.593	0.608	0.619	0.628		0.584	0.602	0.622	0.617	0.611	
Diseases of the musculoskeletal system and connective tissue												
Total number	395	371	360	365	396	0.010	4650	4697	4680	4718	4756	0.189
In the city	234	236	238	244	270		3076	3152	3155	3203	3207	
Rate of comprehensiveness	0.592	0.636	0.661	0.668	0.682		0.662	0.671	0.674	0.679	0.674	
Diseases of the genitourinary system												
Total number	251	266	282	276	247	0.149	2288	2366	2340	2420	2496	<0.001
In the city	133	160	168	157	147		1568	1646	1634	1755	1814	
Rate of comprehensiveness	0.530	0.602	0.596	0.569	0.595		0.685	0.696	0.698	0.725	0.727	
Total												
Total number	4028	4002	4167	3949	4045	<0.001	39,253	39,785	39,226	39,073	38,799	<0.001
In the city	1948	2086	2222	2210	2278		27,398	28,182	27,646	27,833	27,652	
Rate of comprehensiveness	0.484	0.521	0.533	0.559	0.563		0.698	0.708	0.705	0.712	0.713	

## Data Availability

The datasets used and/or analyzed during the current study may be obtained from the corresponding author upon reasonable request.
